# Waiting on the Urine: The Impact of Delayed Urine Collection on Length of Stay in the Emergency Department

**DOI:** 10.1016/j.acepjo.2025.100236

**Published:** 2025-08-12

**Authors:** David Arastehmanesh, Bryan A. Stenson, Anne Grossestreuer, Lakshman Balaji, Peter S. Antkowiak, Leon D. Sanchez, David T. Chiu

**Affiliations:** 1 Department of Emergency Medicine, BronxCare Health System, New York, New York, 10457, USA; 2 Department of Emergency Medicine, Beth Israel Deaconess Medical Center, Boston, Massachusetts, 02215, USA; 3 Department of Emergency Medicine, Brigham and Women’s Faulkner Hospital, Boston, Massachusetts, 02130, USA

**Keywords:** urinalysis, UA, ED length-of-stay, urine collection

## Abstract

**Objectives:**

Efficient collection of urinalyses (UAs) can be variable and is patient-dependent. Anecdotal delays in UA collection, compared with those for blood tests, can potentially lead to prolonged length of stay (LOS) and worsened throughput. Our study sought to analyze the impact of delayed urine collection on emergency department (ED) operations.

**Methods:**

This study is a retrospective cohort analysis of 38,640 discharged encounters with a urinalysis and 67,645 discharged encounters with a complete blood count (CBC). We captured order time, collection time, and disposition time. We calculated deciles of order-to-collection times for UA and CBC groups to visualize the data. We separated the groups into patients with UA only, CBC only, and both CBC + UA. We performed adjusted and unadjusted analyses using Kruskal-Wallis tests and a quantile regression to compare median order-to-collection times as well as to compare the relationship between LOS and order-to-collection time between groups.

**Results:**

Median order-to-collection time in the CBC-only group was significantly shorter than the CBC + UA group (16.7 minutes [IQR, 3.7-40.6] vs 75.6 minutes [IQR, 26.9-150.5]). Median LOS was shortest in the UA-only group (259 minutes [IQR, 187-367]), followed by the CBC group (339 minutes [IQR, 255-449]) and lastly, the CBC + UA group (392 minutes [IQR, 302-507]). In the CBC + UA group, the UA order-to-collection time is a median of 37 minutes (IQR, 0-120) longer than that of the CBC. Each additional minute of order-to-collection time in the CBC-only group contributed 0.50 minutes (IQR, 0.47-0.53) to LOS, compared with 0.58 minutes (IQR, 0.56-0.60) in the CBC + UA group and 0.90 minutes (IQR, 0.83-0.97) in the UA-only group (*P* < 0.001).

**Conclusion:**

UAs take longer to collect than CBCs. Ordering a UA in addition to a CBC significantly increased order-to-collection time and LOS. Each additional minute of collection delay contributed 16% more to LOS in this group, and with higher collection time outlier cases for UAs, this can lead to a worsening bottleneck. Understanding these effects may help EDs prioritize UA collection and target interventions in which there may be the highest impact on flow.

**The Bottom Line**

This study looked to quantify delays in urinalysis collection and analyze the subsequent operational impacts on key time intervals such as length of stay (LOS). Urinalyses took longer to collect at each decile of time to complete blood counts (CBCs). Collecting urinalysis and CBC (vs CBC alone) added a median of 52 minutes to LOS. Each additional minute delay in specimen collection contributed significantly more to the overall LOS. These data suggest delayed urinalysis collection may be a bottleneck for departmental throughput and could help inform where to deploy targeted interventions to improve flow.

## 1 Introduction

### 1.2 Background

Efficient patient throughput is a key operational factor for all emergency departments (EDs). Once patients are seen and evaluated, a medical workup is initiated—potentially including laboratory work or imaging studies—and the subsequent tests need to result before a patient can be discharged or admitted. Delays in obtaining these tests lead to longer disposition times and ED length of stay (LOS).^1^ Prolonged LOS can lead to crowding, and all the negative downstream effects this brings with care delays.^2^, ^3^, ^4^ Additionally, improvements in ED timeliness have been associated with improved patient satisfaction.^5^ Previous studies have looked at potential ways to mitigate these prolonged lengths of stay by enacting front-end operational projects such as triage protocols for urinalysis collection or other standing order sets.^6^^,^^7^

One potential source of these delays is urinalyses (UAs), which are frequently ordered throughout most EDs on a variety of patient complaints. Whether requested by the clinician after patient evaluation or obtained as part of a nursing order set, the interpretation of UA results is often necessary prior to determining patient disposition and can sometimes change the trajectory of a patient’s workup.

Collection of UAs is largely patient-dependent and, as such, poses a different operational challenge compared with samples that are more nursing dependent such as viral swabs or blood tests. There has been research in this area conducted in infants, demonstrating that cutaneous suprapubic stimulation is associated with earlier clean-catch urine collection.^8^ However, adult patients who are young and healthy often can provide a sample of urine on their own and interventions may be more passive. A delay could still occur if patients are not reminded to collect a sample or are not given a specimen cup prior to going to a restroom. One single-center study found no difference in disposition times for ambulatory arrivals with a chief complaint of abdominal or flank pain when given a urine specimen cup upon arrival and encouraged by an ED greeter to provide a sample.^6^ On the other hand, elderly patients and those with significant comorbidities are sometimes unable to provide urine specimens, and therefore, a more collaborative effort among various staff members must be undertaken to assess the patient’s volume status, possible urinary retention, and to help patients void using proper technique to obtain an adequate, “clean-catch” sample. Within the past decade, novel external collection devices such as condom catheters and “PureWicks” have been increasingly used in EDs. These have helped with patient comfort as well as hygiene, but there is limited literature evaluating their ability to collect sterile samples, which are necessary for cultures as well as most UAs.^9^

Although uncollected UAs are often anecdotally noted as a factor delaying ED care, there has been limited research on the true impacts of delayed urine collection on operational metrics such as disposition time and LOS. One specific observational study found an association between ordering of a urinalysis and LOS.^10^ Another agreed that UA ordering increased LOS, but not as much as blood tests.^11^ However, these did not look at the specific time interval from order to urine collection and the effects on LOS. This paper seeks to quantify those effects to see how delayed urine collection may be affecting overall departmental throughput.

### 1.2 Importance

It is critical to understand any potential factors that may prolong ED LOS, as this can affect overall patient care and even impact crowding, which has further downstream effects. Recognizing potential bottlenecks during a patient’s ED stay—and quantifying their impact—may encourage hospital and departmental leadership to deploy resources or new strategies to address this issue. Focused initiatives to systematize urine collection may be more likely to occur once the impact of these delays is demonstrated.

### 1.3 Goals of This Investigation

We have anecdotally observed that UAs take much longer to collect than blood tests at our own institution. Our study aims to assess how order-to-collection times differ between UAs and a less patient-dependent test (complete blood counts [CBCs]). CBCs were chosen as a very common blood test, with similar laboratory turnaround times compared with those of UAs, whose collection typically involves only direct intervention from ED staff and is rarely dependent on patient behavior. Additionally, we evaluate whether delays in the collection of urine are associated with longer ED LOS.

## 2 Methods

### 2.1 Study Design and Setting

This study took place at a northeast, urban, academic, tertiary care center with a yearly volume of approximately 47,780 annual ED visits. The hospital institutional review board approved the study design with a waiver of informed consent.

### 2.2 Selection of Participants

We performed a retrospective review of all adult patients seen in our academic ED from July 1, 2017, to June 30, 2022. We included only patients who were discharged from the ED and excluded those admitted, transferred, or placed into observation as these dispositions may occur prior to the collection of a urinalysis that was ordered.

**Figure 1:**
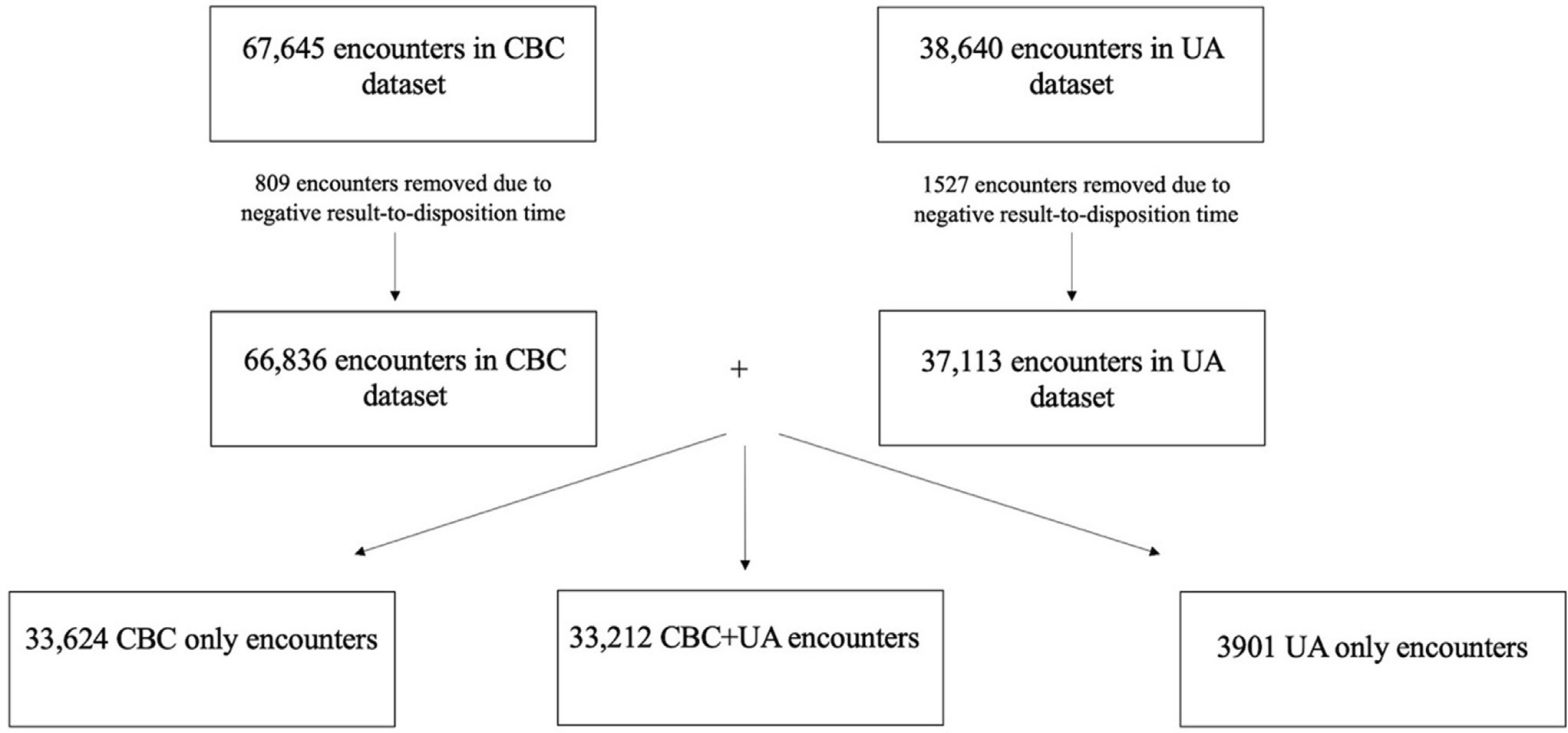
Flowchart for patient group selection. CBC, complete blood count; UA, urinalysis.

As UAs were our test of interest, we needed a reference group with a different test whose order-to-collection time had less variability. We chose CBC for this, as this was the most common blood test ordered, and its collection was less likely to depend on patient behavior and was often only dependent on nursing or phlebotomy workflow. We analyzed data only on patients who had a urinalysis and/or a CBC ordered with available timestamps of order time, collection time, and result time. The data were pulled from the institution’s homegrown electronic health record. Of note, specimen collection time was entered by the nurse when the blood or urine was sent to the laboratory. Patients were excluded if the disposition occurred prior to the results of the UA or CBC. The dataset was separated into 3 groups—encounters with only a CBC ordered, those with only a UA ordered, and those with both (Fig 1).

### 2.3 Measurements

We calculated both UA and CBC order-to-collection times for all cases, as well as the overall LOS. We also tracked the result-to-disposition interval, and cases with a negative value were excluded as these indicate a disposition decision was made without this result. Negative order-to-collection intervals were kept in the dataset as this indicated the blood or urine was collected early on, before the order was even placed.

### 2.4 Exposures, Outcomes, and Analysis

Descriptive statistics were presented on all eligible patients, grouped by whether they had both UA and CBC, UA only, or CBC only. Patient characteristics were described between groups using medians with IQR or counts with proportions. Continuous data were compared using Kruskal-Wallis tests and categoric data using chi-squared tests (Table 1).

**Table 1:** Patient demographics.

Variable	UA and CBC (n = 33,212)	UA only (n = 3901)	CBC only (n = 33,624)	*P* value
Age, median (IQR)	48 (31, 65)	39 (25, 59)	51 (33, 65)	<0.001
Female sex, n (%)	22,853 (69)	2600 (67)	17,771 (53)	<0.001
Race, n (%)				<0.001
American Indian/Alaska Native	65 (0)	10 (0)	77 (0)	
Asian	1980 (6)	264 (7)	1888 (6)	
Black/African American	8198 (25)	1121 (29)	8858 (26)	
Hispanic/Latino	4247 (13)	457 (12)	3672 (11)	
Native Hawaiian/Pacific Islander	43 (0)	4 (0)	42 (0)	
White	16,476 (50)	1742 (45)	16,830 (50)	
Others	2078 (6)	275 (7)	2105 (6)	
Unknown/not specified	125 (0)	28 (1)	152 (0)	
ESI, n (%)				<0.001
1	609 (2)	26 (1)	680 (2)	
2	8,614 (26)	605 (16)	10,550 (31)	
3	23,586 (71)	2755 (71)	21,469 (64)	
4	399 (1)	507 (13)	912 (3)	
5	4 (0)	8 (0)	13 (0)	
LOS (h), median (IQR)	6.5 (5.0, 8.5)	4.3 (3.1, 6.1)	5.7 (4.2, 7.5)	<0.001
Calendar year seen				<0.001
2017	3834 (12)	630 (16)	3023 (9)	
2018	7552 (23)	1073 (28)	6571 (20)	
2019	7165 (22)	846 (22)	6772 (20)	
2020	5435 (16)	460 (12)	5878 (17)	
2021	6402 (19)	554 (14)	7364 (22)	
2022	2824 (9)	338 (9)	4016 (12)	
Academic year seen				<0.001
2017-2018	7643 (23)	1145 (29)	6357 (19)	
2018-2019	7365 (22)	994 (25)	6774 (20)	
2019-2020	5974 (18)	643 (16)	6030 (18)	
2020-2021	6002 (18)	462 (12)	6470 (19)	
2021-2022	6228 (19)	657 (17)	7993 (24)	

CBC, complete blood count; LOS, length of stay; UA, urinalysis.

After excluding negative result-to-disposition values, order-to-collection time was calculated for all of the cases in each group. For the group with both a UA and CBC, the interval was calculated from the first of those laboratory results that were ordered until the last one that was collected.

**Figure 2:**
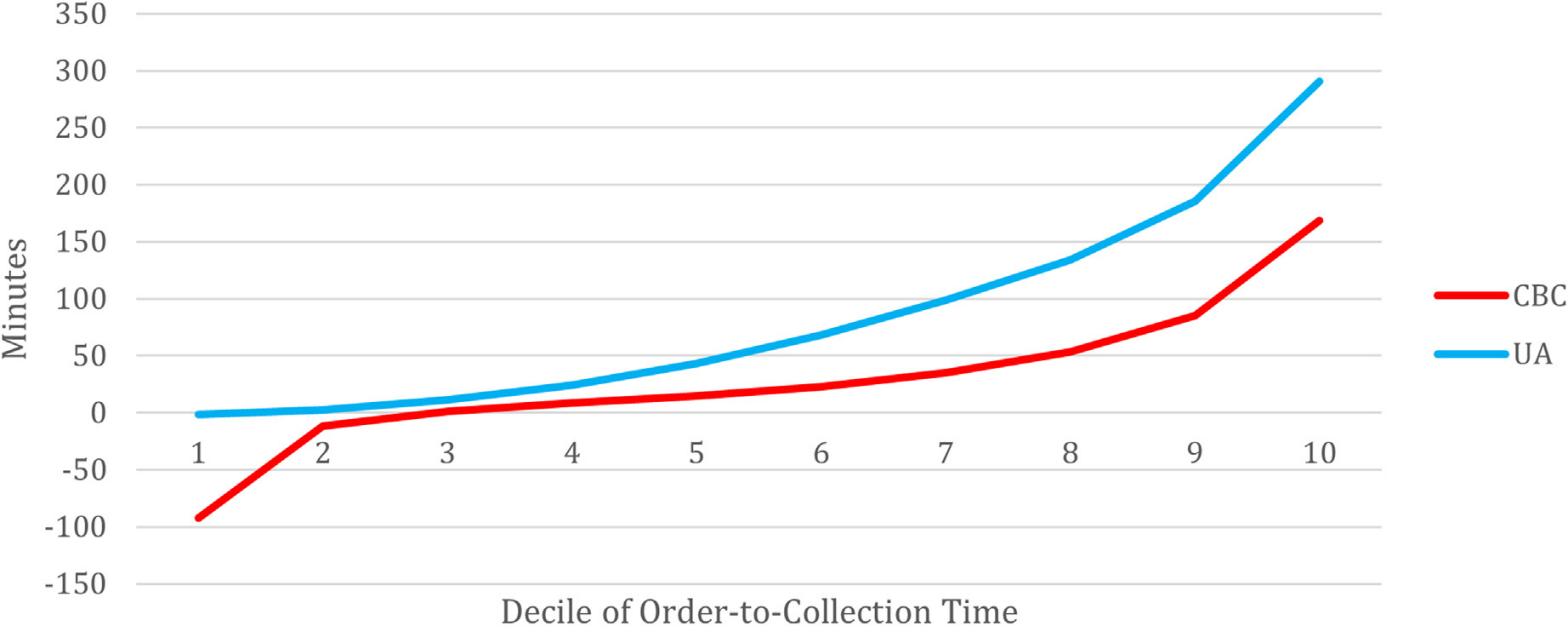
Median observed order-to-collection time for each decile. CBC, complete blood count; UA, urinalysis.

For initial data visualization, we looked at all order-to-collection times pooled together for both UAs and CBCs. We separated them into deciles, which were chosen due to there not being a linear relationship across the timespan, and this allowed us to see how the relationship between order-to-collection times of UAs and CBCs differed at the highest extreme. We graphed the median value for each 10% of the data and displayed it in Figure 2. The specific ranges of each decile can be found in Supplementary Table 1.

To compare order-to-collection time and LOS between groups, we performed both adjusted and unadjusted analyses. The unadjusted analyses compared medians using Kruskal-Wallis tests; the adjusted analyses adjusted for age, sex, and Emergency Severity Index (ESI) and used a quantile regression due to the nonnormality of the data, with the CBC-only group as the reference group.

To compare the relationship of order-to-collection time to LOS, we first ran a quantile regression model including all patients to get the overall median coefficient, adjusting for age, sex, and ESI. We then stratified the analysis by group and ran both unadjusted and adjusted quantile regressions within each group, adjusting for age, sex, and ESI. Finally, we ran a quantile regression with all patients with an interaction term between group and time from order-to-collection time to compare the relationship of LOS and order-to-collection time between groups.

Analyses were performed using either Stata 18.5 (College Station) or R Statistical Software (v4.1.1; R Core Team 2021). Statistical significance was a priori determined as a *P* value of <0.05, and all estimates were provided with 95% CIs.

## 3 Results

We included 38,640 patients who had a urinalysis ordered and 67,645 patients with a CBC ordered. When separating the patients into 3 groups, there were 33,212 patients with both a UA and CBC (CBC + UA) ordered, 33,624 with only a CBC ordered, and 3901 with only a UA ordered.

The groups were statistically different in terms of acuity, with the UA-only group being younger, having less acuity, and less likely to be White. The CBC-only group was less likely to be female compared with the other 2 groups (Table 1).

The median order-to-collection time in the CBC-only group was 16.7 minutes (IQR, 3.7-40.6) and 15.7 minutes (IQR, 2.4-58.0) in the UA-only group, both of which were significantly shorter than the 75.6 minutes (IQR, 26.9-150.5) in the CBC + UA group. The median LOS was significantly shorter in the UA-only group (median 259 minutes [IQR, 187-367]) compared with the CBC-only group (median 339 minutes [IQR, 255-449]) or the CBC + UA group (median 392 minutes [IQR, 302-507]) (Table 2). Within the CBC + UA group, the order-to-collection time for the UA is a median of 37 minutes (IQR, 0-120) and a mean of 59 minutes (SD,123) longer than the order-to-collection time for the CBC.

**Table 2:** Relationship of order-to-collection time and LOS to group.

Group	Median (IQR)	*P* value	Adjusted median difference (95% CI)^a^	*P* value
Order to collection (min)				
CBC only	16.6 (3.7, 40.6)	<0.001^b^	Reference	
UA only	15.7 (2.4, 58.0)		−1.1 (−3.6-1.3)	0.364
CBC + UA	75.6 (26.9, 150.5)		58.7 (57.6-59.8)	<0.001
LOS (min)				
CBC only	339 (255, 449)	<0.001^b^	Reference	
UA only	259 (187, 367)		−66.5 (−72.6, −60.4)	<0.001
CBC + UA	392 (302, 507)		52.3 (49.5-55.1)	<0.001

CBC, complete blood count; LOS, length of stay; UA, urinalysis.

a Controlling for age, sex, and Emergency Severity Index.

b Significant between all groups.

Table 3 demonstrates the relationship of order-to-collection time on LOS. Each additional minute of order-to-collection time in the CBC-only group contributed 0.50 minutes (0.47-0.53) to LOS. This was compared with 0.58 minutes (0.56-0.60) in the CBC + UA group and 0.90 minutes (0.83-0.97) in the UA-only group, a statistically significant difference.

**Table 3:** Relationship of order to collection to LOS.

Overall analysis	Unadjusted coefficient (95% CI)	*P* value	Adjusted coefficient (95% CI)^a^	*P* value
All groups			0.60 (0.58-0.61)	<0.001


Stratified analysis	Unadjusted coefficient (95% CI)	*P* value	Adjusted coefficient (95% CI)^a^	*P* value
CBC	0.50 (0.47-0.53)	<0.001	0.49 (0.46-0.52)	<0.001
UA	0.90 (0.83-0.97)	<0.001	0.82 (0.76-0.89)	<0.001
CBC + UA	0.58 (0.56-0.60)	<0.001	0.57 (0.56-0.59)	<0.001


Model with all groups	Unadjusted median difference (95% CI)	*P* value	Adjusted median difference (95% CI)^a^	*P* value
CBC	Reference		Reference	
UA	0.40 (0.32-0.48)	<0.001	0.35 (0.27-0.43)	<0.001
CBC + UA	0.08 (0.04-0.11)	<0.001	0.08 (0.05-0.11)	<0.001

CBC, complete blood count; LOS, length of stay; UA, urinalysis.

a Controlling for age, sex, and Emergency Severity Index.

Additionally, Figure 2 allowed us to visually compare the median order-to-collection time for both UAs and CBCs. Although no formal statistical tests were performed because of concerns regarding data independence, visual inspection by decile shows that order-to-collection times at the highest deciles were much higher for UAs than CBCs and may be representative of an additional impact as a bottleneck.

## 4 Limitations

Our study was conducted at a single, academic, urban, tertiary center whose results may not be generalizable to the majority of EDs throughout the country or abroad. One reason for this is the variation in operational workflows regarding urine collection. Some sites may give specimen cups out at triage, whereas others may have a policy for performing a catheterization at a distinct time point. This variability limits the external validity.

At our tertiary center, there are many layers of supervision, which may delay time intervals because the student or resident needs to get approval from the attending prior to discharge. Residents are also carrying a high patient census and managing patients in parallel, which can create delays in interpreting the UA results. We also only analyzed patients who were discharged home as opposed to admitted to the hospital, transferred, or placed into observation. Only discharged patients were analyzed as other disposition decisions may occur prior to all the laboratories resulting and would have potentially complicated the analysis. An additional limitation of our study was our inability to control for other variables that could have impacted overall LOS including imaging studies as well as other laboratory tests such as viral swabs and non-CBC blood work.

We were also only looking at workflow optimization and did not focus on patient safety issues. Additionally, our data analysis was based on the collection times noted by the nurse or technician in our electronic health record, but do not know how long the urinalysis may have been produced and waiting for the ED staff to send it to the laboratory.

## 5 Discussion

This study analyzed the relationship between urine collection time and its impact on overall ED LOS in an attempt to identify bottlenecks. Our results demonstrated that the time from order-to-collection of UAs appears longer compared to CBCs. This aligns with anecdotal experience that UAs often take a long time to collect, and the highest deciles of order-to-collection times for the UA appear much larger than the CBC dataset.

For the UA-only dataset, every additional minute of delay in urine collection led to 0.9 minutes of additional LOS. This was significantly higher when compared with the 0.5 minutes of additional LOS for the CBC-only group and 0.58 minutes in the CBC + UA group. The values are lower in the groups with CBCs, suggesting that there are other aspects of care—such as imaging, medication administration, or consultant evaluations—also contributing to the total LOS. The UA-only group is younger and less acute and has overall shorter LOS, and therefore, it is not surprising that any additional wait to collect the UA is likely one of the last steps prior to discharge for these patients.

We also found a lot of value in comparing the CBC + UA group with the CBC-only group, in which both the median order-to-collection time and LOS were significantly higher when a UA was also ordered. Within the CBC + UA group, it was also shown to take a median of 37 minutes longer to collect a patient’s UA versus their CBC. The upper limit of the IQR was 120 minutes, meaning that 25% of this group took over 2 additional hours to collect a urine when both were ordered.

Additionally, the contribution to LOS from a delayed collection for the CBC + UA group was 16% longer than the CBC-only group. The CBC + UA group has a longer LOS at baseline (approximately 6.5 vs 5.5 hours in the CBC-only group), but it is still notable that each additional minute delay in specimen collection adds statistically more time to that group’s LOS. Because there can be significant collection delays for UAs, this can contribute to cases of very prolonged LOS.

Although we only analyzed patients who were being discharged home, these data confirm our experience that delays in urine collection are significant contributors to prolonged LOS. In patients with only a UA ordered versus those with only a CBC ordered, the additional LOS is almost double for every minute delay in collection. Additionally, patients who got both a CBC and UA ordered vs a CBC alone had both prolonged baseline LOS and an increase in the contribution of collection delays on LOS. This suggests that urine collection is a bottleneck in our department and that focusing additional resources to help expedite the collection of urine may decrease overall ED LOS. Even though admitted and transferred patients were excluded, there is likely to be some percentage of admitted patients in which the collection of urine is the bottleneck for either disposition decision or antibiotic initiation, and targeted initiatives may provide some benefit.

Delays in the collection of urine have been a source of frustration for many clinicians working in EDs and is often an item that is heavily nursing dependent. Given the unprecedented increase in ED volumes across the United States as well as the shortages of clinical staff, most notably nurses, it is our hope that this paper will shed light on areas we can focus our already limited resources to help improve ED throughput. Although our analysis described the relationship between order-to-collection times and LOS for these different groups, there may be room to explore the impacts at the extremes of delayed urine collections as these increased greatly for the most delayed groups and may lead to outlier cases. Future studies can assess what causes such delays; we speculate that possibilities include miscommunication between nursing staff and physicians, patients and clinical staff, or poor access to specimen cups throughout the ED. Interventions that may expedite the collection of urine may include the placement of urine specimen cups in all bathrooms in the ED with reminder signs, giving urine specimen cups to patients in triage and waiting room areas, as well as automatic pages or messages to clinical staff if no urine specimen has been collected at predetermined intervals. Additionally, such automatic messages can include the consideration of giving a gentle intravenous fluid bolus, oral fluids, or a one-time urinary catheterization depending on the patient’s comorbidities and hydration status. Given that automatic messages and pages are already instituted in most electronic medical records, such modifications can be highly effective while being low cost. All of these are potential future areas of research to further characterize this issue.

In summary, our data suggest that a delay in urine collection time is a bottleneck in our department and can potentially lead to increased LOS. Urine collection should be prioritized with the same urgency as CBC collection, and focusing our resources to expedite this collection in the ED may potentially improve LOS, overall throughput, and operational metrics.

## Author Contributions

DA: conceptualization, methodology, formal analysis, investigation, and writing—original draft.

BAS: conceptualization, methodology, formal analysis, investigation, writing—original draft, and writing—review and editing.

AG: formal analysis and writing—review and editing.

LB: formal analysis and writing—review and editing.

PSA: writing—review and editing.

LDS: conceptualization, supervision, project administration, and writing—review and editing.

DTC: conceptualization, supervision, project administration, and writing—review and editing.

## Funding and Support

By *JACEP Open* policy, all authors are required to disclose any and all commercial, financial, and other relationships in any way related to the subject of this article as per ICMJE conflict of interest guidelines (see www.icmje.org). The authors have stated that no such relationships exist.

## Conflict of Interest

All authors have affirmed they have no conflicts of interest to declare.
