# Supplementary material for: Waiting on the Urine: The Impact of Delayed Urine Collection on Length of Stay in the Emergency Department
Source: J Am Coll Emerg Physicians Open. 2025 Aug 12;6(5):100236. doi: 10.1016/j.acepjo.2025.100236 (PMC12361769; doi:10.1016/j.acepjo.2025.100236)
Supplement: Supplementary Table 1 [file mmc1.docx]

Supplemental Table 1 – UA and CBC Order-to-Collection Time Deciles

|  | UA Dataset | CBC Dataset |
| --- | --- | --- |
| Decile | Order-to-Collection Time (minutes; range) | Order-to-Collection Time (minutes; range) |
| 1^st^ | Below 0.45 | Below -37.98 |
| 2^nd^ | 0.47, 6.47 | -37.88, -2.17 |
| 3^rd^ | 6.48, 16.78 | -2.15, 5.00 |
| 4^th^ | 16.8, 32.97 | 5.02, 11.35 |
| 5^th^ | 32.98, 55.13 | 11.37, 18.67 |
| 6^th^ | 55.17, 82.97 | 18.68, 28.47 |
| 7^th^ | 83.0, 115.25 | 28.5, 42.88 |
| 8^th^ | 115.27, 156.38 | 42.9, 66.65 |
| 9^th^ | 156.4, 225.72 | 66.67, 113.68 |
| 10^th^ | Above 225.77 | Above 113.7 |
